# Sodium-Glucose Co-transporter-2 Inhibitor-Induced Pruritus: Itching for Answers

**DOI:** 10.7759/cureus.17573

**Published:** 2021-08-30

**Authors:** Kabeer Ali, Saeed R Mohammed, Rishi Deonarine, Surujpal Teelucksingh

**Affiliations:** 1 Internal Medicine, Eric Williams Medical Sciences Complex, Champs Fleurs, TTO; 2 Faculty of Clinical Medical Sciences, University of the West Indies, Trinidad, TTO; 3 Internal Medicine, Medical Associates Hospital, St. Joseph, TTO

**Keywords:** type 2 diabetes, sglt-2 inhibitor, chronic pruritus, maculopapular rash, type 1 diabetes mellitus (t1d)

## Abstract

Sodium-glucose co-transporter 2 inhibitors (SGLT2-I) have revolutionized the treatment of type 2 diabetes mellitus during the last decade. It has not only proven to be very effective for glycemic control but also has adjunctive effects in the management of heart failure, hypertension, and diabetic nephropathy, and even contributes to weight loss. Another benefit is the apparent lack of major side effects, particularly hypoglycemia, apart from euglycemic diabetic ketoacidosis. The most well-known side effects are genital mycotic infections and urinary tract infections (UTI). Although pruritus is less well known, we highlight in this case study this side effect as notable albeit uncommon so as to sensitize clinicians to its possibility.

## Introduction

We present a case of a 47-year-old female with type 2 diabetes mellitus who experienced severe acute pruritus and generalized maculopapular rash upon initiation of dapagliflozin [1]. She experienced the same side effect six months later with empagliflozin. We concluded that the pruritus and rash were due to the SGLT2-I as cessation of the drug on both occasions led to the abatement of symptoms. Through a detailed description of this index case and a summary of a series of other such cases in our practice, we aim to highlight the nonspecific nature of this adverse consequence of SGLT2-i therapy and elucidate its presentation and potentially debilitating side effect.

## Case presentation

A 47-year-old woman presented to the endocrinology clinic with a history of uncontrolled type 2 diabetes mellitus. She was previously being treated with metformin 500 mg daily and gliclazide 80 mg daily. Two weeks prior to presentation, she, of her own accord, increased her dose of metformin to 1 g twice daily and stated her fasting blood glucose (FBS) results were subsequently more stable. Laboratory tests revealed random blood glucose (RBS) of 190 mg/dl (10.6 mmol/L) and glycated hemoglobin (HbA1c) of 10.8% (95 mmol/mol). She was counseled thoroughly on these results and the implications of such a high HbA1c and a therapeutic alliance was established. Gliclazide was discontinued, and dapagliflozin (5 mg daily for one week; 10 mg daily thereafter) and insulin glargine (six units subcutaneously at night) were initiated. She was given a one-week follow-up appointment to review her home blood sugar logs on her new medication regimen.

Two days after the introduction of dapagliflozin, she telephoned the clinic to report that she developed severe pruritus involving the arms, legs, and trunk along with a hyperpigmented maculopapular rash in these same areas. She was advised to discontinue dapagliflozin, which led to the resolution of pruritus, and skin lesions resolved within three days. At her follow-up appointments, she admitted that she experimented with the SGLT2-I and began taking the medication once weekly. On each instance, the pruritus and rash recurred within three days of re-initiation and resolved completely three days after stopping the drug, lending further credibility to the opinion that her symptoms were solely due to the drug.

At a clinic visit three months later, her RBS was 145 mg/dl (8.1 mmol/L) and HbA1c was 9.6% (81 mmol/mol), and she reported that she was no longer experimenting with dapagliflozin. She was switched from metformin 1 g twice daily to a metformin/sitagliptin 50 mg/1000 mg combination twice daily and continued on six units of insulin glargine.

An evaluation after three months revealed an RBS of 150 mg/dl (8.3 mmol/L) and a markedly improved HbA1c at 7.0% (53 mmol/mol). She also reported her FBS now ranged from 80 to 125 mg/dl (4.4-6.9 mmol/L); however, she had resumed taking gliclazide 80 mg once daily. In an effort to wean the patient off of subcutaneous insulin injections, as well as the sulfonylurea, both were discontinued. She was advised to continue metformin/sitagliptin and a trial of a different SGLT2-I, empagliflozin (10 mg daily for one week; 25 mg daily thereafter), was initiated.

One week later, she again experienced similar severe pruritus, observing widespread papular lesions with and surrounding hyperpigmentation, comparable to prurigo nodularis. The same phenomenon of cessation of symptoms was observed on the discontinuation of empagliflozin.

**Figure 1:**
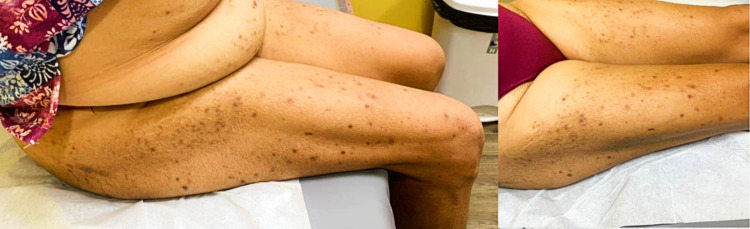
Hyperpigmented scars and papules seen in this patient caused by treatment with sodium-glucose co-transporter-2 inhibitors

**Figure 2:**
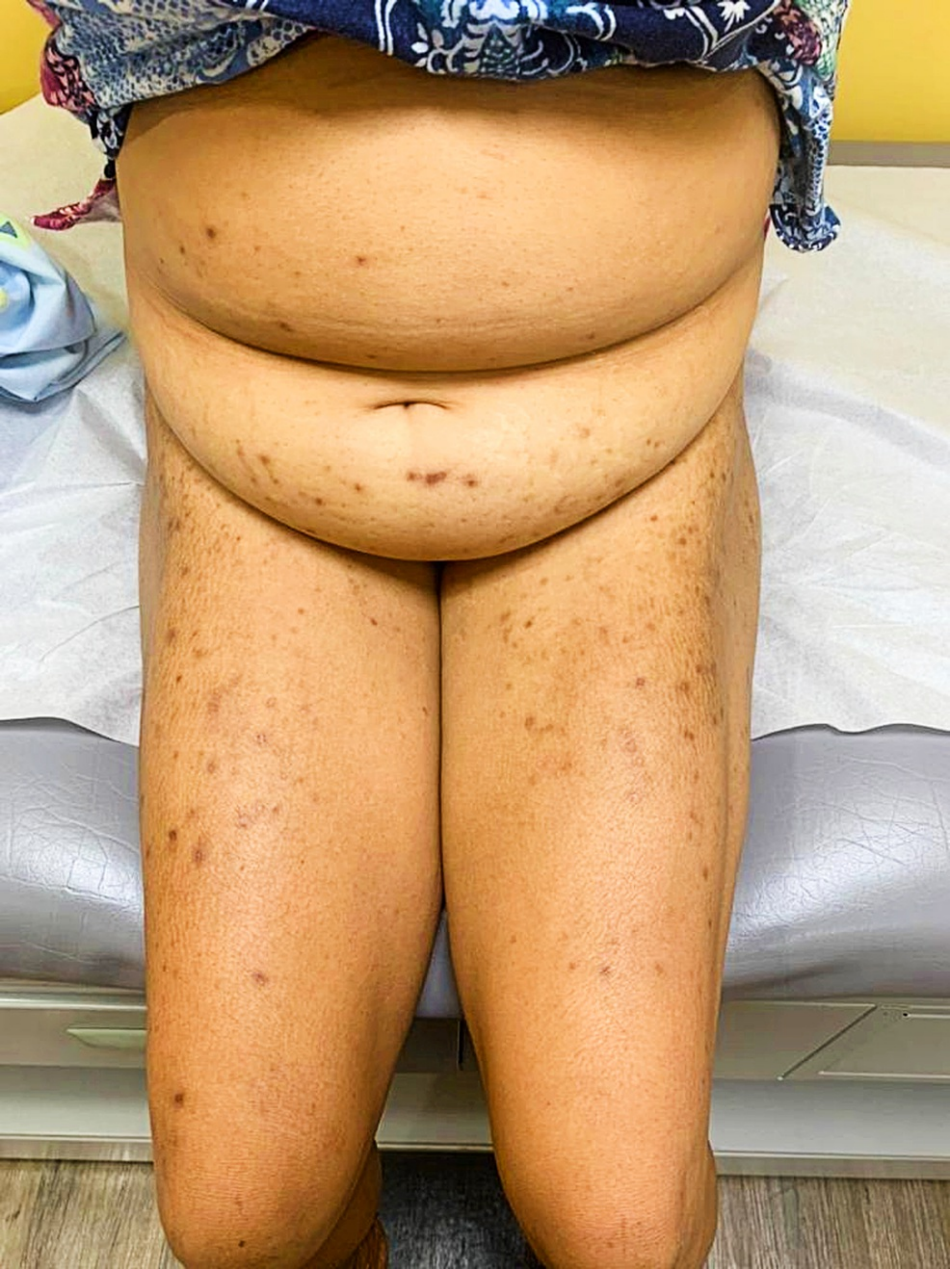
The symmetrical distribution of this patient’s rash is highlighted here

The patient eventually achieved satisfactory glycemic control despite being intolerant of SGLT2-I with just metformin/sitagliptin 50 mg/1000 mg twice daily and adherence to strict diet and exercise. Despite being unable to benefit from the additional effects of SGLT2-I, her diabetes control is improved, and she now enjoys an increased quality of life. Her laboratory values at her most recent clinic visit demonstrated the following: FBS ranging from 90 to 100 mg/dl (5.0-5.6 mmol/L) and HbA1c of 6.4% (46 mmol/mol). However, although the itching has ceased, some hyperpigmented papules and scars persist (Figures 1, 2).

Four additional patients from the endocrinology clinic, where approximately two-thirds of a population of 2500 patients are on SGLT2-I, have since presented with similar symptoms on initiation of SGLT2-I, with resolution on cessation. Unique characteristics of each patient are outlined (Table 1) for comparison purposes.

**Table 1:** Comparison of characteristics of all patients from the endocrinology clinic who presented with SGLT2-I-induced pruritus and rash SGLT2, Sodium-glucose co-transporter 2 inhibitors.

Patient	Age	Sex	Comorbidities	History of Allergy	Drug Used	Naranjo Adverse Drug Reaction Probability Score
1 (Index Case)	47	F	Type 2 Diabetes Mellitus	No	Dapagliflozin and Empagliflozin	7
2	20	F	Type 1 Diabetes Mellitus	No	Empagliflozin	8
3	74	F	Type 2 Diabetes Mellitus, Hypertension, Hypothyroidism, Obstructive Sleep Apnea	No	Empagliflozin	6
4	68	F	Type 2 Diabetes Mellitus, Hypertension, Obesity	No	Empagliflozin	6
5	37	F	Type 2 Diabetes Mellitus, Hypertension, Obstructive Sleep Apnea, Sickle Cell Trait	No	Empagliflozin	7

## Discussion

Drug-induced pruritus is an entity, which is often described, but the underlying mechanism remains elusive. Reich et al. classified this process into acute and chronic - with acute being characterized by pruritus of fewer than six weeks duration and spontaneous resolution after drug cessation and chronic being associated with pruritus of more than six weeks duration and the absence of spontaneous cessation of pruritus after discontinuation of the drug [1]. Using these criteria, our patients had acute pruritus, with a resolution of both pruritus and rash after treatment withdrawal.

In the analysis of this adverse effect of the drug, the authors have looked at several factors: (1) any relevant demographic data, (2) a look at the unique pharmacological properties of SGLT2-I, (3) reviewing other medications known to cause drug-induced pruritus and attempting to establish a connection between those and SGLT2-I, and (4) analyzing any previous reports of SGLT2 inhibitor-induced pruritus.

The authors looked at several possible correlating factors between our affected patients and risk factors for pruritus, and little convincing evidence was found. None had any previous history of food or drug allergies or even atopy to suggest previous exaggerated histamine response. The only consistency was that all our affected patients were women. Our case patient experienced pruritus with both empagliflozin and dapagliflozin with a further dermatologic manifestation, that being a hyperpigmented and maculopapular rash. According to a review of existing literature by Weisshaar et al. on the epidemiology of itching, it is still very poorly understood despite it being the most common presenting symptom in dermatology [2], and thus it is imprudent to draw conclusions from demographic data.

A look into the history of drug-induced pruritus reveals a long list of many groups of drugs causing itch by various pathophysiological mechanisms, with the most common being cholestatic liver injury. These have mainly been examined in individual case reports, and targeted randomized controlled trials have not been conducted [1]. In addition to drug-induced cholestasis, other etiologies of drug-induced itch include it being secondary to pre-existing skin lesions or from an increase in bradykinin level, such as in the case of angiotensin-converting enzyme (ACE) inhibitors.

It is very unlikely that cholestatic liver injury is the mechanism behind SGLT2 inhibitor-induced itch. Acute liver injury from SGLT2-I is very rare, nor has it been associated with other forms of liver injury such as vanishing bile duct syndrome or chronic hepatitis [3]. There has even been evidence of a clinically significant reduction in aspartate aminotransferase (AST) and alanine transaminase (ALT) values in diabetic patients with non-alcoholic steatohepatitis (NASH) when SGLT2-I was introduced, suggesting that SGLT2 inhibitors can possibly be used as adjunctive treatment in non-alcoholic fatty liver disease [4]. Dapagliflozin is the only class of SGLT2-I with a likelihood score relating to “possible rare cause of clinically apparent liver injury” [3]. This is supported by an isolated case report published on dapagliflozin-induced liver injury; however, this occurred in a patient who had pre-existing Child’s Class A cirrhosis [5]. The lack of hepatic metabolism of SGLT2 inhibitors may result in their low incidence of liver injury, with metabolism being mainly through uridine diphosphate (UDP) glucuronosyltransferase [3].

An interesting perspective is the relationship between the renin-angiotensin-aldosterone (RAAS) system and SGLT2 inhibition. RAAS blockade in diabetics is essential to prevent microalbuminuria, the first sign of diabetic nephropathy, and hence most patients with diabetes are initiated on either an ACE inhibitor or angiotensin receptor blocker (ARB), unless contraindicated [6]. The SGLT2 co-transporter is located on the proximal convoluted tubule of the nephron, through which angiotensin 2 exerts its effect to increase sodium reabsorption. A synergistic relationship has thus been shown between RAAS blockade and SGLT2 inhibition, leading to a combined renoprotective effect in diabetics [7].

Particularly in the presence of combination ACE inhibitors and SGLT2 inhibitors, an increase in the ratio of ACE2:ACE was seen according to a study conducted by Cherney et al. [8]. ACE2 counters the effects of ACE and acts as a central negative regulator of the RAAS system. A well-known additional effect of ACE in addition to RAAS activation is its role in the metabolism of bradykinin into inactive metabolites, and we postulate that further ACE inhibition provided by SGLT2-I may propagate increased bradykinin levels to a greater extent. Bradykinin has been shown to be an etiological factor for drug-induced pruritus in relation to ACE inhibitors [1]. However if it is shown to be a potentiating factor for drug itch, practitioners should expect other effects of increased bradykinin, most notably an increase in the incidence of dry cough.

Previous reports of specific SGLT2 inhibitor-induced pruritus have been rare. A case report has shown a 61-year-old woman who developed generalized intense pruritus after initiation with canagliflozin treatment, which resolved after cessation of the drug [9]. In our case report, the Naranjo Algorithm or Adverse Drug Reaction Probability Scale provides a score of 7, which correlates with “Probable Adverse Reaction.” In a three-month post-marketing surveillance report on the short-term impacts of SGLT2 inhibitors in Japanese clinical practice, several reports of generalized rashes and drug eruptions appeared [10]. Ipragliflozin was the first SGLT2 inhibitor to be associated with these reports, but dapagliflozin, tofogliflozin, and luseogliflozin which were later introduced in Japan were also implicated, though with a lower incidence of serious reactions. Severe generalized rash, urticaria, erythema, and eczema were all observed and generally appeared within two weeks of starting treatment, though sometimes even on the first day. This study suggested that such skin reactions were largely specific to ipragliflozin and to a lesser extent, dapagliflozin [10].

Mellander et al. researched over 20 phase IIb and phase III clinical trials of dapagliflozin for the rate and characteristics of hypersensitivity-induced skin adverse events, inclusive of Asian patients, and concluded that "dapagliflozin does not lead to an increased risk of serious hypersensitivity reactions or potentially hypersensitivity-related skin events" [11]. It has also been reported that the incidence of skin rash in East Asian patients was low and comparable between empagliflozin 10 mg and placebo [12]. The incidence rate ratio was numerically higher than one for the 25 mg dose, but no severe skin rashes were reported. However, reports of skin toxicity manifesting as rash, urticaria, or photosensitivity in international pharmacovigilance databases from the WHO and the FDA suggest these reactions are not limited to ethnicity as was earlier thought [13].

SGLT2-I is now a mainstay in the management of diabetes and cardiovascular disease. Skin reactions are rare but may occasionally be severe enough to warrant discontinuation of this useful group of drugs. The mechanism underlying this effect is unclear and requires further investigation. Further randomized control trials to elucidate etiology, prevention, and exploration of useful treatment options for drug-induced pruritus should be undertaken. We reported this case to highlight this under-explored entity that may preclude the use of an otherwise multisystemic beneficial therapeutic agent.

## Conclusions

We presented a case of a 47-year-old type 2 diabetic woman who upon initiation of two different SGLT2-I six months apart developed identical skin reactions with pruritus and rash, and since then we have identified four more cases with similar experiences. Fortunately, this phenomenon is uncommon; however, its mechanism is yet unknown. This provides an opportunity for researchers to explore this infrequent adverse effect seen in one of the most commonly prescribed drugs in today’s practice.
